# Multimorbidity in Severe Mental Illness as Part of the Neurodevelopmental Continuum: Physical Health-Related Endophenotypes of Schizophrenia—A Narrative Review

**DOI:** 10.3390/brainsci14070725

**Published:** 2024-07-19

**Authors:** Vadim Genkel, Elena Domozhirova, Elena Malinina

**Affiliations:** 1 Department of Internal Medicine, South-Ural State Medical University, Chelyabinsk 454092, Russia; 2 Department of Psychiatry, South-Ural State Medical University, Chelyabinsk 454092, Russia; domozhirova-ev@mail.ru (E.D.); malinina.e@rambler.ru (E.M.)

**Keywords:** schizophrenia, neurodevelopment, endophenotype, polygenic risk score, inflammation, cardiovascular disease, multimorbidity, atherosclerosis

## Abstract

Background. The majority of deaths in patients with schizophrenia and other severe mental illnesses (SMIs) are caused by natural causes, such as cardiovascular diseases (CVDs). The increased risk of CVD and other somatic diseases in SMIs cannot be fully explained by the contribution of traditional risk factors, behavioral risk factors, patients’ lifestyle peculiarities, and the influence of antipsychotics. The present review has the following main objectives: (1) to aggregate evidence that neurodevelopmental disorders are the basis of SMIs; (2) to provide a review of studies that have addressed the shared genetic architecture of SMI and cardiovascular disease; and (3) to propose and substantiate the consideration of somatic diseases as independent endophenotypes of SMIs, which will make it possible to place the research of somatic diseases in SMIs within the framework of the concepts of the “neurodevelopmental continuum and gradient” and “endophenotype”. Methods. A comprehensive literature search was performed on 1 July 2024. The search was performed using PubMed and Google Scholar databases up to June 2024. Results. The current literature reveals considerable overlap between the genetic susceptibility loci for SMIs and CVDs. We propose that somatic diseases observed in SMIs that have a shared genetic architecture with SMIs can be considered distinct physical health-related endophenotypes. Conclusions. In this narrative review, the results of recent studies of CVDs in SMIs are summarized. Reframing schizophrenia as a multisystem disease should contribute to the activation of new research on somatic diseases in SMIs.

## 1. Introduction

Schizophrenia is a severe mental illness (SMI) characterized by positive (delusions, hallucinations, and thought disorganization) and negative (alogia, social withdrawal, blunted affect) symptoms, leading to progressive cognitive impairment and social functioning deficits [1,2,3]. According to recent epidemiological studies, the raw prevalence of schizophrenia, incidence, and disability-adjusted life years (DALYs) worldwide has been steadily increasing from 1990 to 2019 [3]. The greatest burden of schizophrenia is observed in the population aged 25–54 years, thus affecting the most productive and economically active part of society [4]. According to various data, the life expectancy of patients with schizophrenia is 15–20 years lower than the average population values, and this gap continues to widen due to the increase in life expectancy in the general population [5]. It should be noted that the majority of deaths in patients with schizophrenia and other severe mental illnesses are caused by natural causes, such as cardiovascular diseases (CVDs), respiratory diseases, infections, and cancer [5,6,7]. The contribution of CVDs to the reduction in life expectancy in schizophrenia and bipolar affective disorder (BAD) is estimated to be about 17.4% in men and 22.0% in women [8]. According to the analysis of a Global Burden of Disease (GBD) study, schizophrenia is associated with an increase in the relative risk (RR) of death from coronary artery disease by 2.36 times (95% UI 1.77–3.14), stroke—by 1.86 times (95% UI 1.36–2.54), and diabetes—by 4.08 times (95% UI 3.80–4.38) [9]. Both schizophrenia and other SMIs, such as BAD and schizoaffective disorder, are associated with an increased risk of adverse cardiovascular events [10]. According to a study by R. Rossom et al. (2022) [10], which included 11,333 patients with SMI, patients with SMI compared to the control group had a significantly higher 10-year cardiovascular risk (9.44% vs. 7.99%) after adjusting for potential confounding factors. Also, among patients with SMI, the proportion of individuals with a high 30-year cardiovascular risk was significantly higher—25% vs. 11% (*p* < 0.001) in the control group. Undoubtedly, traditional cardiovascular risk factors play a crucial role in the increased risk of CVD in patients with SMI. For example, in the study by R. Rossom et al., obesity and smoking made the most significant contribution to the increase in cardiovascular risk. A number of studies have indicated that among patients with SMI, the prevalence of traditional cardiovascular risk factors is quite high, including when compared with the general population and at the onset of the disease before the prescription of antipsychotics [11,12,13]. Additionally, the category of antipsychotic medication might be linked to potential adverse effects of these drugs (K. Uludag et al., 2021) [14]. In contrast, in the study by L.R. Jorgensen et al. (2023), schizophrenia diagnosed within the last 2 years was associated with an increase in the odds ratio of atherosclerotic peripheral artery disease (PAD) after adjustment [15]. This finding is consistent with previously obtained data demonstrating the most pronounced increase in the relative risk of PAD among patients with schizophrenia at a young age (20–34 years) [16].

It should be noted that excess mortality among patients with mental disorders is determined by a broad range of factors, each of which influences the other. There is strong evidence of the essential role of ethnicity, family bonds, and religious identity as determinants of mortality, including in patients with mental illness [17,18,19]. These are the factors that, according to S. Yusuf, can be described as “causes of causes” of diseases [20]. However, it is likely that the increased risk of CVD and other somatic diseases in SMI cannot be fully explained by the contribution of traditional risk factors, behavioral risk factors, patients’ lifestyle peculiarities, and the influence of antipsychotics [21]. It is extremely important that cardiometabolic and respiratory disorders are more often detected in first-degree relatives of patients with SMI without mental disorders compared to the general population [22,23]. This finding may indicate a common genetic architecture of schizophrenia (and other SMIs) and somatic disease, including cardiometabolic diseases. Thus, a number of genome-wide association studies (GWASs) have allowed the identification of common loci underlying the development of schizophrenia and susceptibility to smoking, development of arterial hypertension, glucose metabolism disturbance, dyslipidemia, and coronary artery disease [21,24,25,26,27,28].

Considering schizophrenia and other SMIs as systemic diseases, in which comorbid somatic diseases have a common genetic architecture and etiopathogenetic factors, can contribute to significant progress in studying the mechanisms and features of somatic diseases, including cardiovascular diseases, in SMI, as well as improving approaches to providing multidisciplinary care to patients with SMI [15,21,29].

The present review has the following main objectives: (1) to aggregate evidence that neurodevelopmental disorders are the basis of SMI; (2) to provide a review of studies that have addressed the shared genetic architecture of SMIs and cardiovascular disease; and (3) to propose and substantiate the consideration of somatic diseases as independent endophenotypes of SMIs, which will make it possible to place the research of somatic diseases in SMIs within the framework of the concepts of the “neurodevelopmental continuum and gradient” and “endophenotype”.

A comprehensive literature search was performed on 1 July 2024. The search was performed using PubMed and Google Scholar databases up to June 2024. The search was performed using various combinations of key terms including: «schizophrenia», «cardiovascular disease», «severe mental illness», «neurodevelopment», «atherosclerosis», «GWAS», «endophenotype», «cardiovascular disease», «multimorbidity», and «polygenic risk score». Although this review does not include the methodological requirements of a systematic review, we followed available recommendations on methodology for writing narrative reviews, including the Scale for the Assessment of Narrative Review Articles (SANRA) methodology [30,31].

## 2. Neurodevelopmental Disorders as a Common Mechanism for the Development of Schizophrenia and Other SMIs: The Neurodevelopmental Continuum

### 2.1. Neurodevelopmental Hypothesis of Schizophrenia

Currently, there are many hypotheses on the etiopathogenesis of schizophrenia proposed within the framework of genetic, immunological, neurochemical, neuromorphological, and other areas of biological research in psychiatry [32]. One of the hypotheses of the etiopathogenesis of schizophrenia which is increasingly being confirmed is the neurodevelopmental hypothesis proposed by D.R. Weinberger in 1986 [33]. According to this hypothesis, the occurrence of schizophrenia is the result of the interaction of adverse genetic and environmental factors leading to disruption of neurodevelopment in the perinatal period and the emergence of symptoms at a later age [34]. One variant of this hypothesis is the “double hit” model, according to which brain development disorders in the perinatal period create a vulnerable substrate for a “second hit” occurring in another period of critical brain development, namely adolescence, when synaptic pruning processes, decreased synaptic plasticity, and myelination of the prefrontal cortex are observed [27]. It should be noted that the neurodevelopmental hypothesis is not isolated and can be successfully integrated with neurochemical and inflammatory hypotheses [35,36].

To date, the results of the largest GWAS identified 287 independent genetic risk loci for schizophrenia [37]. A significant proportion of these loci are associated with neurodevelopment in the perinatal or postnatal periods and represent pathways associated with synapse formation in early developmental stages, neurite growth and axon/dendrite branching, synaptic pruning, synapse density formation, and microglial synaptic engulfment [34]. Another large cluster of genes associated with schizophrenia is a cluster of at least 61 genes also associated with changes in brain macrostructure that can be visualized by magnetic resonance imaging of the brain (brain surface area, cortical thickness, neurite density index) [38]. Moreover, genes associated with brain surface area and cortical thickness also demonstrated involvement in neurodevelopmental processes, which led the authors to conclude that genetic associations with structural brain phenotypes likely represent the effects of genetically controlled programs during gestational and early postnatal cortical development.

### 2.2. Neurodevelopmental Disorders as a Common Mechanism of SMI

Furthermore, neurodevelopmental disorders are likely a common mechanism for a number of mental disorders and are not exclusive to schizophrenia. Thus, in a study conducted within the framework of the Cross-Disorder Group of the Psychiatric Genomics Consortium in 2019, it was found that eight mental disorders (anorexia nervosa, attention-deficit/hyperactivity disorder, autism spectrum disorder, bipolar disorder, major depression, obsessive compulsive disorder, schizophrenia, and Tourette syndrome) are characterized by a substantial overlap of genetic substrates [39]. For example, 109 loci were common to at least two mental disorders, and 23 loci were common to four or more disorders [39]. Moreover, one locus, demonstrating the greatest pleiotropy and associated with all eight disorders, is directly related to neurodevelopment and early white matter development, and a loss-of-function mutation in this gene leads to the emergence of a severe neurodevelopmental syndrome with loss of midline commissural tracts and diffuse white matter disorganization [39]. In a later study by A.D. Grotzinger et al. (2022), the common genetic architecture of 11 SMIs was studied at the biobehavioral level, functional genomics levels, and molecular genetics level: attention-deficit/hyperactivity disorder (ADHD), problematic alcohol use, anorexia nervosa, autism spectrum disorder, anxiety disorders, bipolar disorder, major depressive disorder, obsessive compulsive disorder, post-traumatic stress disorder, schizophrenia and Tourette syndrome [40]. The authors were able to identify four broad groups of factors (neurodevelopmental, compulsive, psychotic, internalizing) that underlie genetic correlations between 11 SMIs. Undoubtedly, the common genetic architecture of SMI and the commonality of the main factors of their development should lead to the fact that various endophenotypes (see below) of schizophrenia will have a cross-nosological character and will be observed in a number of SMIs. Confirmation of this was found by Y. L. Chien et al. (2022), who investigated the integrity of white matter tracts at the whole-brain level in patients with schizophrenia, autism spectrum disorders, and ADHD [36]. Patterns of impairment in white matter tract integrity were similar across the three disorder groups, with schizophrenia being indistinguishable from autism spectrum disorders and ADHD when using machine learning algorithms based on this feature. In addition, as expected, the commonality of neuroimaging endophenotypes directly translated into the commonality of cognitive impairments across the three disorders [41].

Thus, on the one hand, neurodevelopmental disorders are genetically determined, underlie the development of SMIs, and are common to them; on the other hand, environmental factors associated with an increased risk of schizophrenia and SMI also largely realize their effects at various vulnerable stages of neurodevelopment, leading to its disruption. The neurodevelopmental hypothesis was expanded upon and developed through the concept of M.J. Owen and M.C. O’Donovan’s “neurodevelopmental continuum” [42,43]. Briefly, SMIs, including schizophrenia, are viewed as reflecting diverse consequences arising from disruption or deviation in brain development. At the same time, childhood neuropsychiatric disorders (intellectual disability, autism, ADHD) and adult SMIs are not viewed as etiologically discrete units but as being on a single etiological and neurodevelopmental continuum or spectrum [43]. A later development of the neurodevelopmental continuum concept is the neurodevelopmental gradient hypothesis [41]. According to this hypothesis, SMIs are arranged within the neurodevelopmental continuum on a gradient of decreasing degrees of neurodevelopmental disorders, as follows: intellectual disability, autism spectrum disorders, ADHD, schizophrenia, and BAD. The place that a disease occupies on this continuum and, accordingly, the gradient of disease severity are determined by factors such as age at disease onset, severity of cognitive impairments, and persistence of functional disorders [42,43].

## 3. The Neurodevelopmental Continuum and Endophenotypes of Schizophrenia: Heterogeneity of Schizophrenia

### 3.1. Neurodevelopmental Continuum and Endophenotypes of Schizophrenia

Schizophrenia and other SMIs are characterized by high heterogeneity of the genetic substrate within each disease [44]. In this regard, the spectrum of schizophrenia can also be viewed as a kind of continuum within the general continuum of neurodevelopment, the space of which reflects the phenotypic heterogeneity of schizophrenia, as determined by the characteristics of the genetic substrate and cumulative polygenic burden [45,46]. In turn, this will determine the heterogeneity of the disease in a broad sense, including the variability of schizophrenia endophenotypes at various levels: morphological, neurochemical, and neuropsychological level.

It is worth briefly mentioning that in the classical definition of the concept of endophenotype given by I.I. Gottesman and T.D. Gould, endophenotype is designated as a measurable component, invisible to the naked eye, located between the disease and the distal genotype [47,48]. Currently, an endophenotype is defined as a genetically determined, measurable indicator with inter-individual variability consistent with the variability of a higher-order target trait (for example, normative function or a particular disorder) [49].

**Figure 1:**
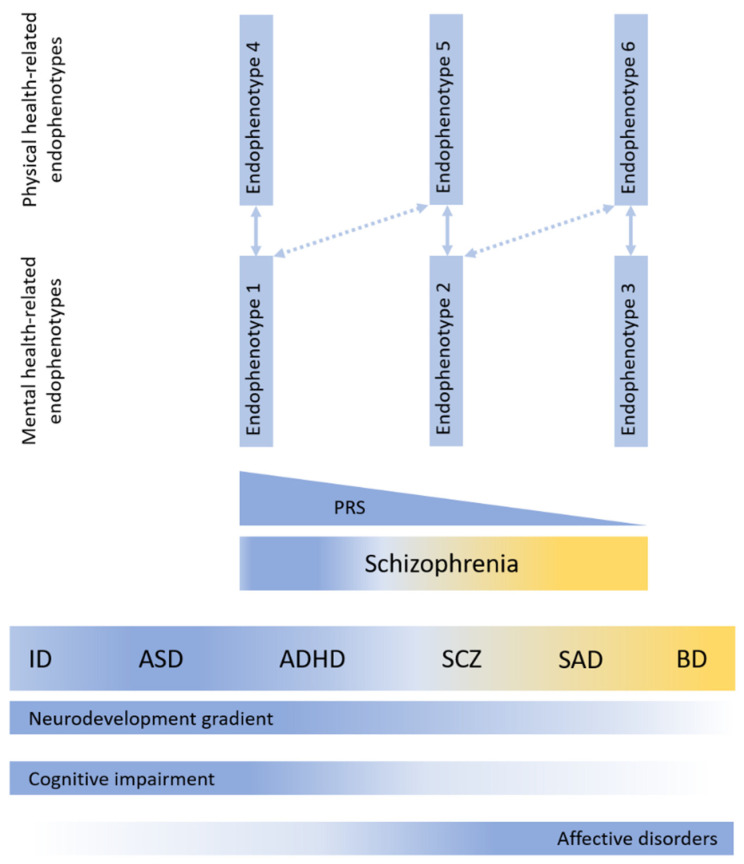
Schizophrenia endophenotypes as a function of location on the neurodevelopmental continuum and polygenic risk score of schizophrenia (ID, intellectual disability; ASD, autism; ADHD, attention deficit/hyperactivity disorder; SCZ, schizophrenia; SAD, schizoaffective disorder; BD, bipolar disorder) adapted from [43,55].

Undoubtedly, the genetically determined heterogeneity of schizophrenia endophenotypes is confirmed in clinical studies. For example, pronounced heterogeneity in various neuromorphological indicators obtained by neuroimaging is well known among patients with schizophrenia, in particular in the thickness and area of the cerebral cortex, ventricular volume, and hippocampal morphology [50,51]. An analysis of the UK Biobank patient cohort confirmed that a greater polygenic risk score of schizophrenia is closely associated with a decrease in the thickness of the frontal and temporal cortex, as well as a smaller hippocampal volume [50]. In another study also performed on the UK Biobank patient cohort, a greater polygenic risk score of schizophrenia was associated with a decrease in the neurite density index at the whole brain level, in 149 cortical regions, 5 subcortical structures, and 14 white matter tracts [52]. In turn, neuromorphological features, such as the area of the frontal and temporal cortex, determine neuropsychological features, such as cognitive ability and psychosocial functioning [53]. At the same time, it is obvious that certain clusters of SNPs included in the complex polygenic risk score for schizophrenia can determine the development of certain endophenotypes (morphological, neuropsychological, etc.), including somatic ones [54]. It is also likely that certain clusters of SNPs associated with the involvement of certain biological pathways are capable of determining the development of several endophenotypes at different levels—anatomical, neurochemical, etc., which in turn leads to the formation of linked endophenotypes of schizophrenia. A potential consequence of this is that by identifying one of the endophenotypes located more or less distally on the genotype–endophenotype–symptom path and appearing more convenient for diagnosis in clinical practice, one can predict with high probability the presence of other clinically significant endophenotypes (see Figure 1).

### 3.2. Physical Health-Related Endophenotypes and Their Interrelationships with the Neuromorphological and Neurophysiological Endophenotypes of Schizophrenia

As an example of such linkage of endophenotypes, we can cite the relationships between retinal changes and neuromorphologic and cognitive status in schizophrenia. According to a meta-analysis of 44 clinical studies, patients with schizophrenia differ from control patients in thinning of the peripapillary retinal nerve fiber layer, macular ganglion cell layer, and retinal thickness in all other macular segments [56]. In addition, peripapillary retinal nerve fiber layer thickness was inversely correlated with the severity of positive and negative symptoms. Moreover, a decrease in the thickness of the peripapillary retinal nerve fiber layer was associated with a decline in cognitive function in both patients with schizophrenia and their healthy relatives [57,58]. In turn, retinal changes in patients with schizophrenia can represent a neuromorphological endophenotype with a decrease in gray matter volume in the occipital and temporal lobes of the brain [59]. These endophenotypes are closely correlated with the polygenic risk score of schizophrenia and are likely associated with pathways of accelerated biological aging and neuroinflammation [60,61,62].

Another example is changes in the parasympathetic nervous system in patients with schizophrenia and their relationships with other traits. Decreased connectivity of the anterior cingulate cortex with the cerebellum and decreased amplitude of low-frequency fluctuations in the cerebellum are the most important neuromorphological and neurophysiological correlates of autonomic nervous system dysfunction in patients with schizophrenia, as assessed by reduced heart rate variability (HRV) [63]. In addition, impaired connectivity of the anterior cingulate cortex with cortical, limbic, striatal, and cerebellar regions is a transdiagnostic feature of SMI, which is characteristic of both schizophrenia and BAD [64]. The decrease in HRV observed not only in patients with schizophrenia but also in healthy relatives of patients indicates a potential genetic substrate for dysregulation of the autonomic nervous system in SMI [65]. Among the genes associated with decreased vagal modulation of heart rhythm and decreased HRV in patients with schizophrenia, we can note *HCN1* (Hyperpolarization-Activated Cyclic Nucleotide Gated Potassium Channel 1), *CACNA1C* (Calcium Voltage-Gated Channel Subunit Alpha1 C), and *KCNH2* (Potassium Voltage-Gated Channel Subfamily H Member 2) [66,67]. It is important to note that polymorphisms in these genes, affecting the functioning of potassium and sodium channels, are associated with both the risk of schizophrenia and the risk of heart rhythm disorders [66,67]. Voltage-gated ion channels play an important role not only in cardiac activity but also in the regulation of neuronal excitability, synaptic plasticity, and rhythmic activity of neurons in brain regions relevant to schizophrenia (the prefrontal cortex and hippocampus) [66]. Decreased HRV and dysregulation of the autonomic nervous system are also correlated with the neurocognitive sphere in patients with schizophrenia. Thus, strong negative correlations have been established between HRV and the severity of psychotic symptoms, as well as cognitive impairments in patients with schizophrenia [68,69,70].

A number of authors have considered the decrease in vagal tone to be an independent endophenotype of schizophrenia [71]. There is an alternative view, according to which the decrease in parasympathetic influences is a manifestation of another endophenotype of SMI: a deficit in inhibitory cognitive control which is manifested, among other things, by a decrease in vagal tone and oculomotor disorders [72]. Undoubtedly, it is debatable whether these disorders represent two independent endophenotypes of schizophrenia or whether they are manifestations of one endophenotype (in this case, a deficit in inhibitory cognitive control) in various physiological, cognitive, and social domains of schizophrenia.

## 4. Cardiovascular Diseases and Schizophrenia: Comorbidity or Systemic Manifestations

### 4.1. Cardiovascular Disease and Schizophrenia: Evidence of a Common Genetic Background

The neurodevelopmental hypothesis focuses exclusively on the brain as the site of ontogenesis disruption [29]. At the same time, it is counterproductive to ignore the systemic disorders observed in schizophrenia, which have a common genetic architecture and represent developmental disorders of the organism as a whole, not just the brain [29]. In fact, the involvement of various organ systems is immanent to schizophrenia and other SMIs and represents separate physical health-related or somatic endophenotypes of the disease.

Despite relatively limited data, there is compelling evidence of both a common genetic substrate and direct causal links between schizophrenia and cardiometabolic risk factors, immune disorders, and structural and functional disorders of the cardiovascular system. H.C So et al. (2019) established polygenic associations between schizophrenia and glucose metabolism disorders (an increase in fasting glucose and insulin), lipid metabolism disorders (an increase in triglycerides), increased leptin, decreased adiponectin, and increased visceral fat [28]. However, the results of Mendelian randomization confirmed direct causal links only between the polygenic risk of schizophrenia and an increase in triglycerides.

Recent studies have revealed changes in the structure and function of the heart in patients with schizophrenia. For example, E.F. Osimo et al. (2020), based on the results of cardiac MRI analysis, found a decrease in heart chamber volumes (a sign of concentric remodeling of the left and right ventricles) in patients with schizophrenia compared with controls after adjusting for age, sex, ethnicity, body surface area, blood pressure, smoking, physical activity level, and medication use [73]. Moreover, according to the analysis of the UK Biobank study, the polygenic risk score of schizophrenia was associated with a decrease in heart chamber volumes (mainly the right ventricle), an increase in ejection fraction, and an increase in myocardial stiffness (i.e., a decrease in peak absolute strain) [74]. Analysis of possible mediators of the relationship between the polygenic risk score of schizophrenia and increased myocardial stiffness demonstrated the potential role of TGF-β (Transforming Growth Factor-β) signaling and inflammatory pathways. The relationships between schizophrenia and structural heart changes also translate into the development of clinically significant endpoints. For example, a study by R.R. Veeneman et al. (2022) using Mendelian randomization showed that genetic predisposition to schizophrenia causally determines the development of heart failure [75]. Later studies have confirmed this relationship [76,77].

### 4.2. Immune System Activation as a Common Pathway for Schizophrenia and Cardiovascular Diseases

It is likely that one of the most important mediators of the development of systemic manifestations of schizophrenia is the immune system; changes have been established at different levels in many studies. A number of GWASs have established a common genetic substrate for schizophrenia and some immunoinflammatory diseases such as Crohn’s disease, ulcerative colitis, primary biliary cirrhosis, and psoriasis [78,79]. To date, more than 30 MHC-related and unrelated genes are known to increase the risk of schizophrenia and represent various immunoinflammatory pathways: *HLA* (human leukocyte antigen), *ARHGAP4* (Rho GTPase-Activating Protein 4), *ARNTL* (aryl hydrocarbon receptor nuclear translocator-like protein 1), *BTG1* (BTG Anti-Proliferation Factor 1), *CD14*, *CD46*, *DOCK4* (dedicator of cytokinesis 4), *EP300* (histone acetyltransferase p300), *FES* (FES proto-oncogene, tyrosine kinase), *FLII* (flightless I actin-binding protein), *FMR1* (fragile X messenger ribonucleoprotein 1), *GCA* (grancalcin), *GNL3* (G protein nucleolar 3), *HBEGF* (heparin-binding EGF-like growth factor), *HSPD1* (Heat Shock Protein family D member 1), *IK* (IK cytokine), *IRAK1* (interleukin-1 receptor-associated kinase 1), *MCL1* (induced myeloid leukemia cell differentiation protein), *MSH6* (MutS homolog 6), *NUCB2* (nucleobindin 2), *PIK3C2A* (phosphatidylinositol-4-phosphate 3-kinase C2 domain-containing alpha polypeptide), *PLA2G15* (phospholipase A2 group XV), *PLCB2* (phosphoinositide phospholipase C-beta-2), *PRKCD* (protein kinase C delta), *PRMT1* (protein arginine methyltransferase 1), *PTGS2* (prostaglandin-endoperoxide synthase 2), *STAT6* (signal transducer and activator of transcription 6), *TPR* (translocated promoter region), *ZEB2* (zinc finger e-box binding homeobox 2), *IFITM1* (interferon induced transmembrane protein 1), *GBP1* (guanylate-binding protein 1), *BST2* (bone marrow stromal cell antigen 2), *IFITM3* (interferon induced transmembrane protein 3), *GBP2* (guanylate-binding protein 2), *CD44* (CD44 molecule), *FCER1G* (Fc epsilon receptor Ig), *FCGR2A* (Fc gamma receptor IIa), *IFI16* (interferon gamma inducible protein 16), and *FCGR3B* (Fc gamma receptor IIIb) [78,80,81]. According to various data, the most relevant inflammatory pathways associated with the development of schizophrenia include *TGF-β*, *TNFR1* (Tumor Necrosis Factor Receptor Superfamily Member 1A), *TOB1* (Transducer of ERBB2, 1), antigen processing, T-cell adhesion molecules, B-cell activation, and CXCL8-related pathways [80,82]. C. Wang et al. (2023) conducted studies with Mendelian randomization, in which causal relationships between 731 immune cell phenotypes and the risk of developing schizophrenia were tested [83]. Among the studied immunophenotypes, only four showed significant relationships with the risk of schizophrenia: CD4^+^ T cells and T regulatory lymphocytes, HLA-DR on monocytes, and CD33^dim^ HLA-DR^+^CD11b^−^ (their increase reduced the risk of schizophrenia). In other studies with Mendelian randomization, bi- or unidirectional genetically determined relationships between schizophrenia and the number of circulating lymphocytes, eosinophils, monocytes, and neutrophils were also shown [84]. Clinical studies have also established persistent neuroinflammation with microglial activation in patients with schizophrenia, as well as activation of innate and adaptive immunity when examining other tissues and peripheral blood markers [85,86,87,88,89,90,91]. Moreover, it is assumed that patients with pronounced activation of the inflammatory response at different levels and in different biological compartments may represent a separate cluster or subtype of patients who are likely to have more pronounced neuromorphological, cognitive, and psychotic disorders and possibly a greater burden of multimorbidity [85,88,92,93,94,95].

### 4.3. Accelerated Biological Aging Is a Common Mechanism for the Development of Schizophrenia and Cardiovascular Diseases

Another possible and critically important mediator of systemic manifestations of schizophrenia is accelerated biological aging, which is often considered an independent endophenotype of schizophrenia and other SMIs. Thus, according to the analysis of 26 international cohorts of patients with schizophrenia conducted by the ENIGMA Schizophrenia consortium, the age of the brain (predicted based on MRI metrics compiled into a brain age prediction model trained on healthy volunteers) in patients with schizophrenia is on average 3.55 years higher than their chronological age (95% CI 2.91–4.1) compared to controls matched for sex and age (in previously conducted studies, these values were higher—from +5.5 to +7.8 years) [96,97]. Accelerated brain aging was also confirmed when assessing the molecular age of the brain in patients with schizophrenia, as evaluated by the expression of 68 and 76 age-dependent genes [98]. Another widely used method in biological age research based on determining epigenetic aging was also applied to cohorts of patients with schizophrenia and BAD. For example, A.P.S. Ori et al. (2024) determined accelerated aging (Levine clock) in later adulthood in patients with schizophrenia compared with controls, regardless of disease duration; it was most pronounced in women with a high polygenic burden (+3.82 years (95% CI 2.02–5.61) [99]. According to a number of authors, the accelerated aging of various organ systems, whose ontogenesis is disrupted along with the ontogenesis of the nervous system, underlies various somatic disorders in schizophrenia [100]. It should be noted that transcriptome-wide association studies have allowed us to establish that disorders in the expression of certain genes associated with schizophrenia are observed not only in brain tissues, but also in the skin, intestines, lungs, pancreas, subcutaneous fat, heart, etc. (i.e., not only in tissues developing from the ectoderm like the nervous system and skin) [101,102,103].

### 4.4. Schizophrenia and Atherosclerosis: Available Data and Future Research Directions

From the information presented above, we can assume at least several pathways, in addition to behavioral features and antipsychotic therapy, that can play a proatherogenic role in schizophrenia: (1) a common genetic substrate of schizophrenia and cardiometabolic RF; (2) genetically determined activation of innate and adaptive immunity; and (3) accelerated biological aging.

There is evidence of peripheral and central vascular dysfunction in patients with schizophrenia: some genes associated with schizophrenia are involved in maintaining vascular homeostasis and post-ischemic repair; signs of blood–brain barrier and neurovascular unit damage in schizophrenia; increase in biomarkers associated with vascular damage and vascular inflammation (S100B, MMP-9, VEGF, ICAM-1, VCAM-1) in cerebrospinal fluid and blood; signs of impaired angiogenesis (down-regulation of CSF1Ri in the brain and spleen); and a pathological pattern of gene expression in endothelial cells of patients with schizophrenia [104,105,106,107]. The results of a meta-analysis of 215 studies, including 13,952 patients with schizophrenia and 10,969 control patients, confirmed an increase in the concentration of circulating cytokines and inflammatory markers in the peripheral blood of patients with schizophrenia, whose role in atherogenesis has also been proven (IL-1β, IL-6, IL-8, IL-10, TNF-α, and CRP) [108]. In turn, in a recent study by Y. Zhu et al. (2023) found that an increase in the concentration of inflammatory cytokines (IL-6, IL-1β, IL-18, IL-8) in the peripheral blood of patients with schizophrenia was associated with the perivascular accumulation of CD163^+^ macrophages, surrounding vessels of small, medium, and large diameter [109]. It cannot be excluded that this vascular inflammation is part of neuroinflammation in schizophrenia; however, it cannot be excluded that this reflects of the systemic effect of the general pro-inflammatory status of patients. A number of authors have suggested that SMI and schizophrenia are based on “inflammaging”, which is triggered by developmental disorders [110].

Clinical data on atherosclerosis in schizophrenia are limited. In the already mentioned study by L.R. Jorgensen et al. (2023), the frequency of PAD detection did not significantly differ from that in the control group. At the same time, schizophrenia established no later than two years ago was independently associated with an increase in the odds ratio of PAD [15]. It should be noted that the median age of patients in the control group and patients with schizophrenia established no later than two years ago was about 24 years. At the same time, the method chosen by the authors for diagnosing atherosclerosis of the lower limb arteries (ankle–brachial index) allows the diagnosis of only hemodynamically significant stenoses, which, in our opinion, is not an optimal choice of method for diagnosing subclinical atherosclerosis.

In several studies, the intima–media thickness (IMT) of the carotid and/or femoral arteries was evaluated as a marker of subclinical atherosclerosis in patients with schizophrenia. In a study by S. Baykara et al. (2020), which included 28 patients with schizophrenia, statistically significantly higher values of IMT of the carotid and femoral arteries were found compared to controls [111]. In the work of H. Bohman et al. (2020), adolescents with early-onset psychosis and BAD differed significantly in intimal thickness according to high-resolution ultrasound (22 MHz)—0.132 mm versus 0.095 mm (*p* < 0.001) [112]. In the study by O. Imre et al. (2023), patients with schizophrenia compared with the control group had significantly higher carotid IMT as well as hematological inflammatory indices (NLR, MLR, PLR, SII, CRP, ESR) [113]. At the same time, an increase in carotid IMT was associated with worse cognitive status, which was assessed using the Montreal Cognitive Assessment Scale. An increase in carotid IMT in patients with schizophrenia has also been reported in other studies [114]. However, IMT is not an adequate marker of subclinical atherosclerosis, and the assessment of various indicators of atherosclerosis burden in future studies is a promising approach for studying the prevalence of atherosclerosis in patients with SMI [115].

## 5. Findings, Limitations, and Future Directions

According to the neurodevelopmental hypothesis, the etiopathogenesis of schizophrenia is based on brain development disorders in the prenatal and early adolescent periods, which arise as a result of the interaction of genetic and environmental factors [34,101]. At the same time, increasing data from clinical, epidemiological, and genetic studies indicate that developmental disorders in schizophrenia are not limited to the brain alone but affect all organ systems to varying degrees, making schizophrenia a systemic disease [29]. In the present review, we have attempted to provide a rationale for the view that somatic diseases in SMIs can be considered as independent endophenotypes of SMIs. Moreover, the association of different endophenotypes of schizophrenia, including somatic endophenotypes, can probably be considered as a function of location on the neurodevelopmental continuum and the polygenic risk score of schizophrenia. Cardiovascular diseases in conditions of impaired ontogenesis, the high burden of mosaic mutations, activation of various biological pathways, and accelerated biological aging are likely characterized by features of course and drivers of progression that differ from the general population.

The main limitation of this review is the unsystematic data collection and the narrative style of this article. This may have contributed to a systematic error in the selection of articles supporting the main considerations in the review. One of the main assumptions of the review is the acceptance of the neurodevelopmental hypothesis of schizophrenia. However, despite the neurodevelopmental hypothesis currently being one of the leading hypotheses, it may underestimate the importance of other factors in the pathogenesis of schizophrenia, such as neurodegeneration [116,117,118].

Another important assumption underpinning the review is the consideration of schizophrenia as a systemic disorder, which at present is also only a hypothesis and requires further research. Research in this field is extremely limited, but few studies support this hypothesis, demonstrating that the severity of multisystem impairments correlates closely with the magnitude of central nervous system disorders [119,120]. Studies with Mendelian randomization and GWASs demonstrating the shared genetic architecture of SMIs and somatic diseases, which we provide in our review, can also be seen as an argument in support of this concept. On the other hand, the selection of these studies may also have been subject to systematic error, and their results should be viewed through the lens of the limitations of GWASs and studies with Mendelian randomization. Thus, for example, summary statistics from well-powered GWASs were only available for individuals of European ancestry [40,75,76,83]. Another limitation of the studies that use polygenic risk scores is that the variance in schizophrenia explained by the polygenic risk score is modest, and small effects could have been missed [74].

The following promising directions for further research can be suggested: (1) investigation of CVD and atherosclerosis in relation to neuromorphological, neurophysiological, and other endophenotypes of schizophrenia; (2) long-term follow-up studies to establish illness-specific drivers of atherosclerosis and CVD in antipsychotic-naïve patients with schizophrenia; (3) exploring genetic predictors of somatic diseases in patients with schizophrenia in multi-ethnic patient groups; and (4) studying the role of immune response and accelerated aging in the development and progression of atherosclerosis and CVD in patients with SMI and schizophrenia.

## 6. Conclusions

According to general opinion, barriers and challenges in the system of care for patients with SMI exist at the levels of patients, medical workers, and the healthcare system as a whole [13]. Fragmentation and lack of coordination between healthcare providers, among other things, underlie the suboptimal assessment of cardiovascular risk in patients with schizophrenia, resulting in inadequate correction of cardiovascular risk factors and high rates of cardiovascular morbidity and mortality among patients with schizophrenia [8,9,12,13,121]. Reframing schizophrenia as a multisystem disease and somatic diseases as independent endophenotypes of SMI should contribute to the activation of new research on somatic diseases in SMI, focusing the attention of specialists providing care to patients on somatic diseases of patients with SMI [122].
